# Caregiving in the USA -- A First Look at State Level Findings

**DOI:** 10.1093/geroni/igaf122.1189

**Published:** 2025-12-31

**Authors:** Terri Guengerich

**Affiliations:** AARP, La Plata, Maryland, United States

## Abstract

For the first time in the history of the National Alliance for Caregiving and AARP’s Caregiving in the US survey, the survey sample size was increased radically to oversample caregivers at the state level. The purpose of these oversamples was to obtain the prevalence of caregivers in each state and to provide more detailed information about caregivers and their experiences at the state level. Information shared during this session will include the sample plan and weighting strategy as well as the prevalence of caregivers at the state level. In addition, summary information will be provided about caregivers and their experiences at the state level.

